# Low-Temperature Direct PECVD Synthesis of Graphene on Si(100) with Increased Methane Flow: Structure and Photoelectric Properties

**DOI:** 10.3390/mi17070801

**Published:** 2026-06-30

**Authors:** Vidmantas Kumža, Rimantas Gudaitis, Asta Guobienė, Andrius Vasiliauskas, Šarūnas Meškinis

**Affiliations:** Institute of Materials Science, Kaunas University of Technology, K. Baršausko 59, LT-51423 Kaunas, Lithuania; rimantas.gudaitis@ktu.lt (R.G.); asta.guobiene@ktu.lt (A.G.); andrius.vasiliauskas@ktu.lt (A.V.)

**Keywords:** microwave PECVD, low-temperature graphene synthesis, Raman scattering spectroscopy, AFM, CAFM, photoelectric properties

## Abstract

Graphene was directly synthesized on monocrystalline Si(100) at 500 °C by microwave plasma-enhanced chemical vapor deposition using an increased CH_4_/H_2_ gas flow ratio. Raman analysis revealed spectral features and intensity ratios consistent with the growth of hydrogenated graphene and revealed changes in defect structure, graphene layer number, and self-doping. Atomic force microscopy measurements showed that the surface morphology and local conductivity strongly depended on the growth conditions. The electrical and photoelectrical characteristics of graphene/Si junctions were correlated with the Raman parameters and surface morphology. For the hydrogenated graphene samples synthesized at 500 °C, the photocurrent, short-circuit current, and open-circuit voltage were found to be competitive with those of pristine graphene reference samples grown at 700 °C. The results demonstrate the potential of low-temperature direct PECVD synthesis for graphene/Si optoelectronic devices.

## 1. Introduction

Graphene is a highly promising material for optoelectronic applications because of its exceptional electrical, optical, and mechanical properties, including high carrier mobility, optical transparency, mechanical flexibility, and chemical stability [1,2,3,4,5,6]. Depending on the synthesis methods and conditions, it can be obtained in different structural forms, such as planar graphene flakes, vertical graphene, and hydrogen-modified graphene-based films [7,8,9,10]. For device applications, planar graphene is particularly attractive; however, its practical implementation is still limited by fabrication challenges. In this case, the exfoliation can provide graphene with low defect density, but it is not suitable for scalable manufacturing because of poor reproducibility and limited throughput [11]. For large-area synthesis, chemical vapor deposition (CVD) on catalytic metals followed by transfer to the target substrate is widely used [10,12]. However, the transfer step complicates the process and may introduce contamination, wrinkles, ripples, and other structural imperfections into the graphene layer [13,14]. Direct graphene growth on semiconducting and dielectric substrates is therefore an attractive alternative for device-oriented applications. In this context, plasma-enhanced chemical vapor deposition (PECVD) is especially promising because it enables graphene formation at substantially lower temperatures than conventional thermal CVD, reduces energy consumption, and improves compatibility with thermally sensitive substrates and various device architectures [15,16].

Remote-plasma PECVD configurations are commonly employed for graphene synthesis to limit plasma-induced damage and excessive etching of the growing graphene layer, thereby promoting the formation of planar graphene films [16]. In contrast, direct-plasma PECVD systems are more difficult to regulate because the growing material is more strongly exposed to plasma effects. Nevertheless, such systems have demonstrated the ability to deposit both planar and vertical graphene architectures [17,18,19,20]. Earlier studies have also shown that, when direct plasma growth is performed on semiconducting and dielectric substrates, the orientation of graphene flakes can be controlled at temperatures of 700–800 °C [17,19,20]. Direct low-temperature graphene synthesis on technologically relevant semiconductor substrates by plasma-assisted methods has already been demonstrated in some studies. In particular, direct graphene synthesis on monocrystalline silicon at 500–550 °C was reported in [21,22]. However, in those studies, the mechanism enabling graphene growth at such a reduced temperature was not clarified.

This study investigates the possibility of reducing the temperature required for direct graphene synthesis on monocrystalline silicon by increasing the methane-to-hydrogen gas flow ratio. This approach enabled a reduction in graphene growth temperature from 700 °C to 500 °C. Concurrently, the dominant defect type shifted from grain boundary defects to hydrogen-related on-site defects. Importantly, systematic optimization of PECVD growth parameters and graphene structure resulted in key photoelectric characteristics, including photocurrent, short-circuit current, and open-circuit voltage, for layers synthesized at 500 °C that are comparable to those of the pristine graphene reference samples grown at 700 °C used in the present study. To elucidate the origin of these effects, graphene samples synthesized with varying CH_4_ and H_2_ gas flows were analyzed using Raman spectroscopy, and representative samples were examined by atomic force microscopy. The relationships between the electrical and photoelectrical properties of the graphene/Si junctions, Raman spectral parameters, and surface morphology were systematically analyzed.

## 2. Materials and Methods

Samples were grown on monocrystalline Si(100) (Sil’tronix Silicon Technologies, Archamps, France). Before synthesis, the substrates were annealed and cleaned using hydrogen plasma to remove any surface contaminants and prepare them for graphene growth. The graphene was synthesized using plasma-enhanced chemical vapor deposition (PECVD) system Cyrannus (Innovative Plasma Systems (Iplas) GmbH, Troisdorf, Germany). Employing this method, a gas mixture of methane (CH_4_) and hydrogen (H_2_) was used. Table 1 contains the synthesis parameters for each sample. During the deposition, a protective enclosure was placed on the substrate to prevent direct plasma exposure [20,23,24]. The graphene synthesis experiments were planned based on our previous study of low-temperature graphene synthesis on thermal SiO_2_ films [23]. In that study, graphene samples grown at 700 °C and 500 °C using 50 sccm methane and 50 sccm hydrogen flows showed very similar Raman spectra and were both identified as hydrogenated graphene. Accordingly, in the present work, the 700 °C samples were used only as reference samples, while the effect of the gas-flow conditions was analyzed primarily within the 500 °C series.

Graphene-silicon diode structures are made by depositing additional metal electrodes. Cr/Cu films are deposited on the graphene through a mask with 500 μm circular holes using vacuum evaporation. The chromium interlayer thickness was 20 nm, and the copper layer was 200 nm thick. The back contact (Al film) is formed on the other side of the substrate. In this way, multiple graphene/Si diodes were fabricated on the same graphene sample. The graphene–silicon diode structure formed in this way enables the efficient fabrication of a graphene/Si(100) Schottky-type contact and is suitable for further comparative investigation of the electrical and photovoltaic properties in the present work.

The graphene–silicon diode structure formed in this way enables the efficient fabrication of a graphene/Si(100) Schottky-type contact and is suitable for comparative investigation of the electrical and photovoltaic properties of the samples prepared in the present work.

Structural properties were studied using Raman spectroscopy (Renishaw inVia Raman spectrometer (Renishaw, Wotton-under-Edge, UK)). Several points were measured on each sample. The results were gathered using a 532 nm green excitation laser. After the scan, the main examined peaks were the D, G, and 2D. A Lorentzian function was used to fit those peaks and extract the full-width at half-maximum (FWHM), peak position, and intensity values. The intensity ratio of the 2D and G Raman peaks (I(2D)/I(G)) is used to determine the number of graphene layers [25]. The intensity ratio of the D and G Raman peaks (I(D)/I(G)) is applied to estimate the defect density [26,27]. The intensity ratio of the D and D‘ Raman peaks (I(D)/I(D’))was used to evaluate the dominating defect type [28,29]. The positions of the G and 2D peaks are used to determine the doping level and mechanical stresses [30,31].

The surface morphology and contact current (representing the electrical conductivity of the samples) were analyzed using a NanoWizard III atomic force microscope (JPK Instruments, Bruker Nano GmbH, Berlin, Germany) in conjunction with conductive AFM (C-AFM) methodology. For atomic force microscopy (AFM) investigations, Pt/Ir-coated silicon probes (CS Instrument, Berlin, Germany) were employed, featuring a coating thickness of 25 ± 5 nm on both the reflex and tip sides. The probe characteristics included a spring constant of 2.7 N/m, a resonant frequency of 60 kHz, a tip radius of curvature of approximately 30 nm, and a pyramidal tip geometry. Images were acquired over 2 µm × 2 µm areas, and the resulting data were processed using JPKSPM Data Processing software (version spm-4.3.13). To ensure measurement accuracy in conductive atomic force microscopy (CAFM), silver (Ag) electrodes were fabricated on the graphene layer. All measurements were conducted under ambient conditions, with the applied bias voltage ranging from −10 to 10 mV. The recorded current noise level was 55 fA.

The electrical properties of samples are studied by measuring the current–voltage (I–V) characteristics. Measurements were performed for five diodes on each sample to assess potential diode-to-diode dispersion. To study the photoelectric properties, the characteristics were recorded in three modes: in the dark (no illumination), ultraviolet (406 nm LED), and infrared (800 nm LED). During measurements, the voltage range was from −2 to +2 V. The current supplied to the LEDs was selected to have the same optical power (5.2 mW) in different measurement modes. The diode properties were evaluated by analyzing current–voltage (I–V) characteristics in the dark, including the reverse current at 0.3 V. The photoelectric parameters—photocurrent (I_ph_), short-circuit current (I_SC_), and open-circuit voltage (U_OC_)—were determined from I–V curves measured under illumination. Mean values and standard deviations were calculated from the measurements of multiple diodes fabricated on the same sample.

## 3. Results

### 3.1. Raman Spectra Analysis

**Figure 1:**
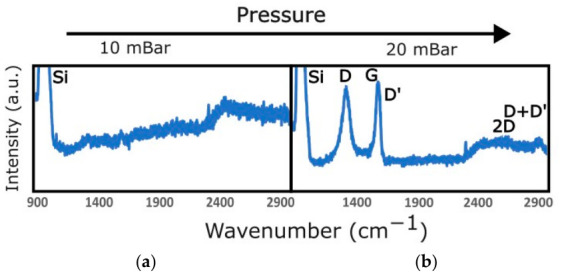
Raman scattering spectra of the graphene samples grown at 500 °C using different work pressures: 10 mBar (**a**) and 20 mBar (**b**).

**Figure 2:**
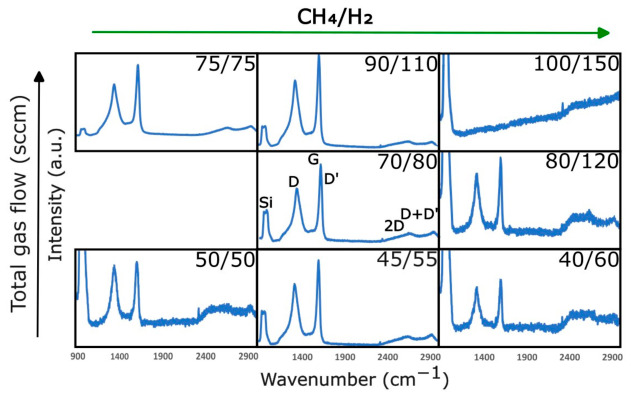
Raman scattering spectra of the samples synthesized using different CH_4_ and H_2_ gas flows at 500 °C. The work pressure in all cases was 20 mBar.

At first, the effects of process pressure were considered. It was revealed that no graphene or other carbonaceous film was grown using 10 mBar work pressure at 500 °C (Figure 1). An increase in the work pressure up to 20 mBar resulted in the successful synthesis of the graphene (Figure 1). Similar behavior was reported in our previous study on low-temperature synthesis of graphene on thermal SiO_2_ films [23]. After establishing the work pressure necessary for graphene synthesis at 500 °C, the CH_4_/H_2_ gas flow ratio and total gas flow were varied to further control the growing layer structure, using a 20 mBar deposition pressure. Representative Raman scattering spectra of the synthesized samples are presented in Figure 1 and Figure 2. Peaks typical for graphene, such as G peak [32,33], D peak [32,33], D’ peak [32,33], 2D peak [32,33], and D + D’ peak [34,35,36,37,38], can be seen in the spectra. In the present samples, the fitted G-band position was in the range of 1596–1604 cm^−1^, while the fitted 2D-band position was in the range of 2598–2670^−1^. It should be mentioned that D, D’, and D + D’ peaks are defect-related [32,33,34,35,36,37,38]. The second-order silicon substrate-related peak in the ~930–990 cm^−1^ range was also observed [39,40].

**Figure 3:**
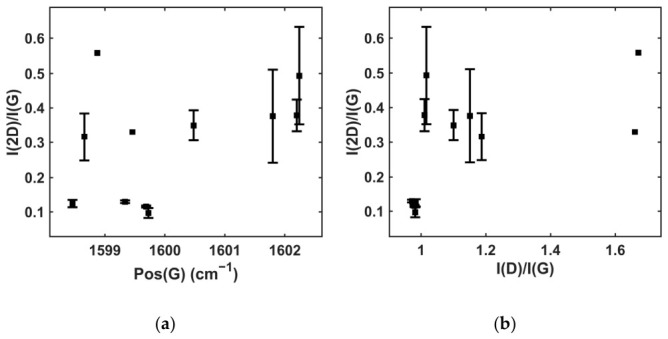

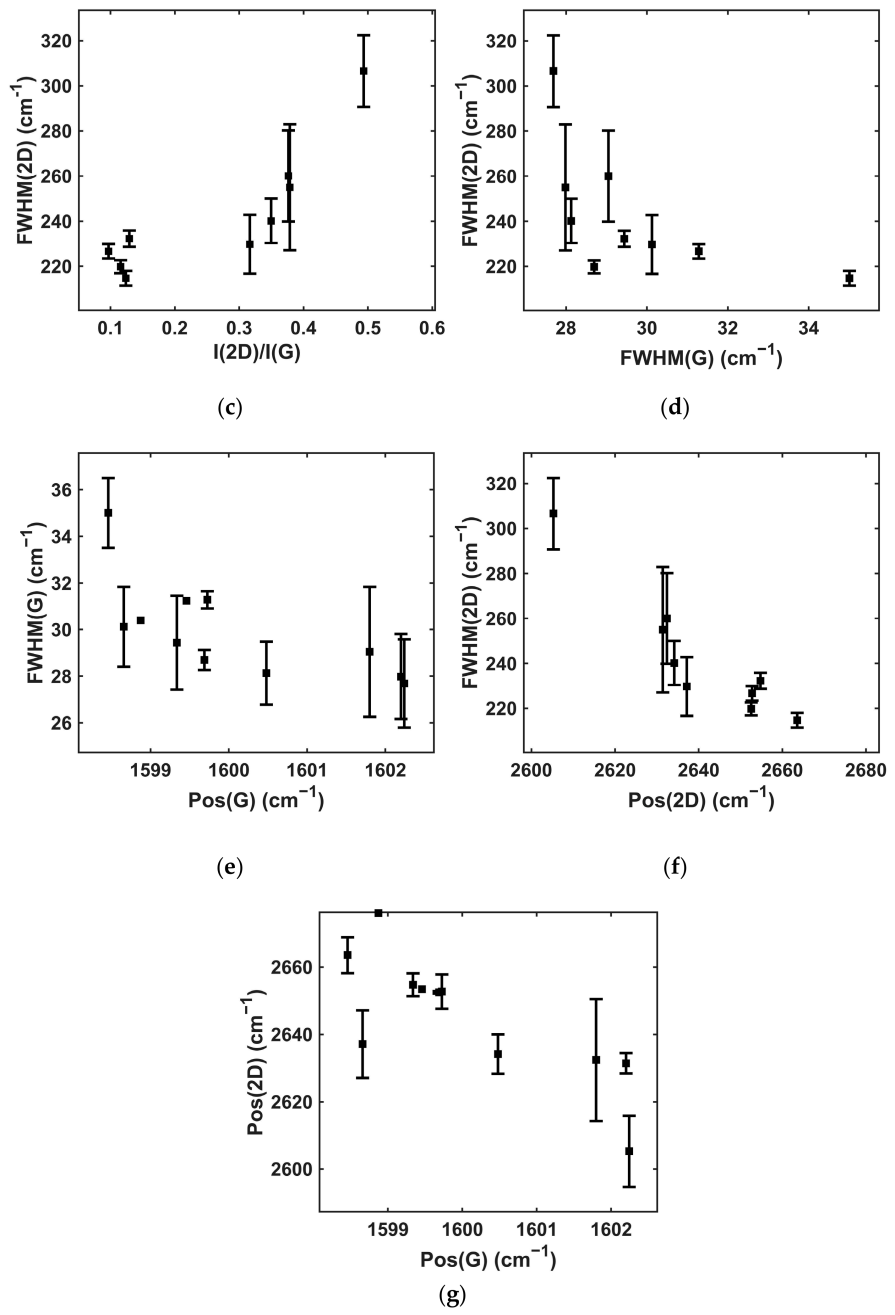
Plots of the Raman scattering spectra parameters: I(2D)/I(G) vs. Pos(G) (**a**); I(2D)/I(G) vs. I(D)/I(G) (**b**); FWHM(2D) vs. I(2D)/I(G) (**c**); FWHM(2D) vs. FWHM(G) (**d**); FWHM(G) vs. Pos(G) (**e**); FWHM(2D) Pos(2D) (**f**); Pos(2D) vs. Pos(G) (**g**). Error bars correspond to Raman parameters dispersion observed within the same graphene specimen.

Graphene Raman-scattering spectra were analyzed. Some Raman spectral parameters of graphene can depend on several factors. Notably, along with the graphene layer number influence, the I(2D)/I(G) ratio can be affected by graphene doping, and in such a case, it should decrease with Pos(G) upshift [31,41]. However, in our case, the tendency is rather opposite (Figure 3a). Thus, in our case, changes in the I(2D)/I(G) ratio are not affected by the doping of the graphene. The I(2D)/I(G) ratio also decreases with increasing defect density [27,42]. However, in the present study, no I(2D)/I(G) ratio decrease with increased I(D)/I(G) ratio was found (Figure 3b). Thus, taking into account the above-mentioned observations, we used the 2D and G peaks intensities ratio to qualitatively estimate the number of graphene layers. FWHM(2D) can become broader with increased graphene layer number [13]. In such a case, FWHM(2D) should decrease with the increase in the I(2D)/I(G) ratio. In Figure 3c, the opposite trend is observed. The 2D and G peaks became narrower with Pos(G) upshift (Figure 3d,e). Also, FWHM(2D) clearly decreased with Pos(2D) upshift. Thus, graphene doping effects can be supposed [31,41]. Pos(2D) downshifted with Pos(G) upshifting (Figure 3f). Such behavior is typical for graphene’s n-type doping [30,31]. In our previous studies, it was attributed to the substrate-induced doping effects [17,20,23,24]. The trend of the I(D)/I(D’) ratio increase with defect density was revealed (Figure S1). The I(D)/I(D’) ratio in all cases was in the ~0.97–2 range. This indicates the prevalence of the on-site defects usually assigned to adsorbed hydrogen atoms [29]. One can see that in the Raman spectra of all of the samples synthesized at 500 °C, the intensity of the 2D peak was the same or even lower than the intensity of the D + D′ peak. Furthermore, the D + D′ peak is associated with the absorption of hydrogen atoms onto the graphene [34,35,36,37,38]. Thus, the growth of the hydrogenated graphene may be supposed [34,35,36,37,38]. However, for samples exhibiting a higher I(D)/I(D’) ratio, the existence of the grain boundary defects along with the adsorbed hydrogen atoms can be supposed [28,29]. No clear dependency of the Pos(2D) on the I(D)/I(D’) ratio was revealed, indicating that graphene defect type has no influence on graphene doping (Figure S1b). Surprisingly, in our previous study on the direct low-temperature synthesis of graphene on thermal SiO_2_ films using high methane flows [23], on-site defects promoted graphene doping. The abovementioned relationships revealed how Raman spectral parameters can be used to analyze structural parameters, graphene layer number, defect type, density, and self-doping.

**Figure 4:**
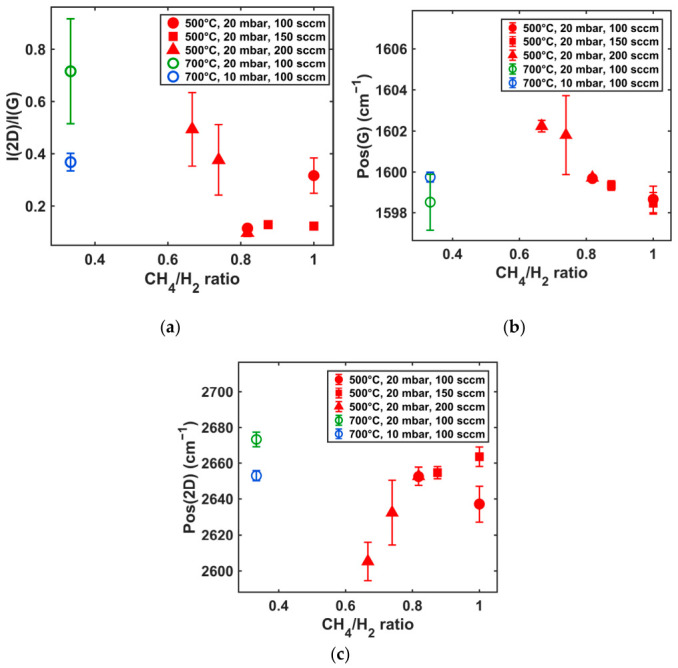
The dependence of the I(2D)/I(G) ratio (**a**), Pos(G) (**b**) and Pos(2D) (**c**) on methane and hydrogen gas flow ratios.

The effects of graphene synthesis conditions were studied by analyzing the dependence of the sample’s Raman scattering parameters on the CH_4_/H_2_ gas flow ratio and the total gas flow used for graphene deposition. No reliance of the defect’s density and prevailing defect type on the abovementioned parameters can be seen in Figure S2a,b. However, it is clear that the I(D)/I(G) ratio of the samples grown with higher methane gas flows was much lower than that of the “pristine” graphene (Figure S2). In these samples, on-site defects clearly prevailed, unlike pristine nanocrystalline graphene, which showed a dominance of boundary defects. The tendency for the graphene layer number to decrease with increasing hydrogen-to-methane gas flow ratio was revealed (Figure 4a). It can be explained by hydrogen-induced etching and reduced flow of the carbonaceous species necessary for graphene growth [43,44]. The trend of the Pos(G) downshift and Pos(2D) upshift with the CH_4_/H_2_ ratio was found for samples synthesized at 500 °C (Figure 4b,c). While no total gas flow effects were revealed, the Pos(G) of the pristine samples was less upshifted compared to most samples synthesized using higher methane gas flows at 500 °C (Figure 4b). The Pos(2D) of pristine samples was higher than in the case of the layers deposited at 500 °C (Figure 4c).

### 3.2. AFM and CAFM Study

**Figure 5:**
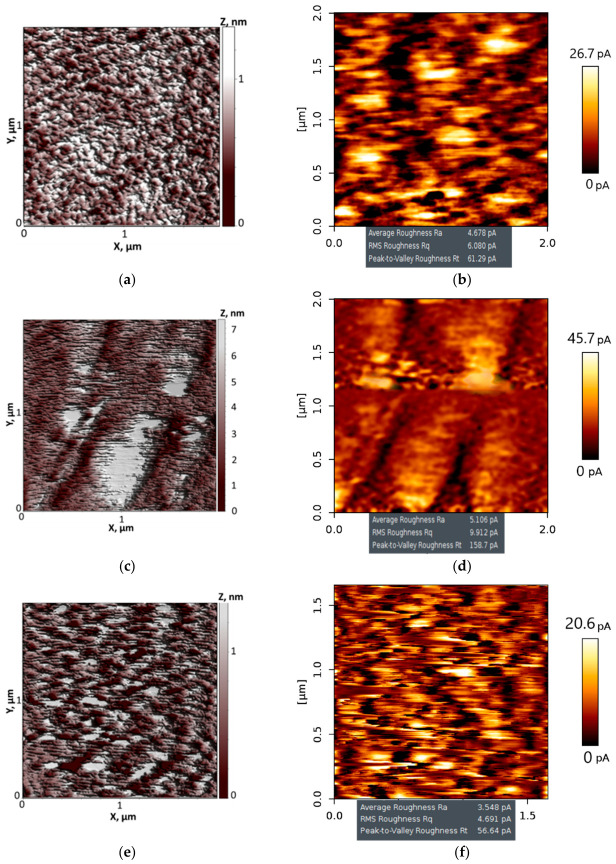
AFM topography (**a**,**c**,**e**) and C-AFM current (**b**,**d**,**f**) maps of graphene layers grown on an n-type silicon substrate at different CH_4_:H_2_ gas flow ratios: 25:75 (**a**,**b**); 80:120 (**c**,**d**); 90:110 (**e**,**f**).

The AFM images in Figure 5 reveal substantial differences in the surface morphology of the analyzed layers. The smoothest surface was observed for pristine graphene (Figure 5a), for which the root mean square roughness (R_q_) was only 0.17 nm. The corresponding skewness value, R_sk_ = 0.08, is close to zero, indicating an almost symmetric height distribution without pronounced topographic extremes. The mean local current measured by conductive AFM was 26.7 pA, suggesting a relatively stable local electrical response across the scanned area.

The results indicate a direct correlation between the gas flow composition and the resulting layer roughness, height, and conduction current parameters (Table 2).

In the case of the hydrogenated graphene synthesized at 500 °C using 80 sccm CH_4_ and 120 sccm H_2_ gas flows, a drastic increase in roughness is observed (R_q_ = 1.59 nm), along with the highest skewness coefficient (R_sk_ = 0.75). This indicates a surface rich in sharp “peaks” or wrinkles. The largest current (45.7 pA) is achieved under these conditions. This may be explained by the “wrinkled” morphology increasing the effective contact area or creating tunneling pathways through defects, which facilitate current flow across the Schottky barrier [45].

The graphene sample grown using increased methane gas flow (110 sccm H2 and 90 sccm CH4) exhibited a much smoother surface, with R_q_ closer to the pristine graphene (0.33 nm). At the same time, the skewness became negative (R_sk_ = −0.09). This indicates that flat terraces dominate the surface, with a minimal number of sharp protrusions (wrinkles). The current decreases and stabilizes at 20.6 pA. Together with the features of the Raman spectra, such as low I(2D)/I(G) ratio and low relative intensity of the substrate-related peak at ~930–960 cm^−1^, this suggests the formation of a continuous, possibly thicker, layer.

### 3.3. Electrical and Photoelectrical Properties

No clear dependence of the dark reverse current (I_rd_) measured at −0.3 V on Raman scattering spectra parameters was revealed (Figure S3). However, the leakage current in the pristine graphene sample synthesized at 700 °C using 20 mBar work pressure was much higher compared to the other samples.

**Figure 6:**
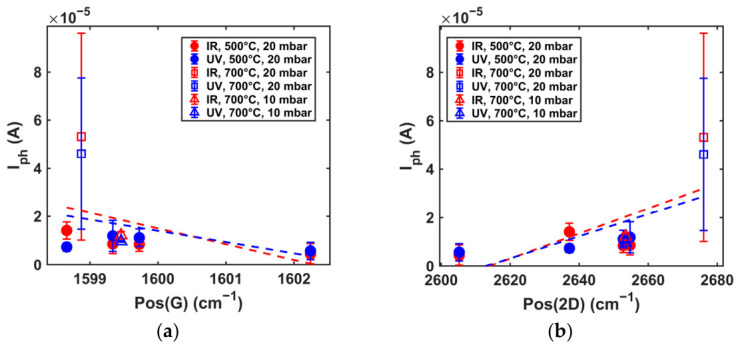
Photocurrent vs. Pos(G) (**a**) and Pos(2D) (**b**).

**Figure 7:**
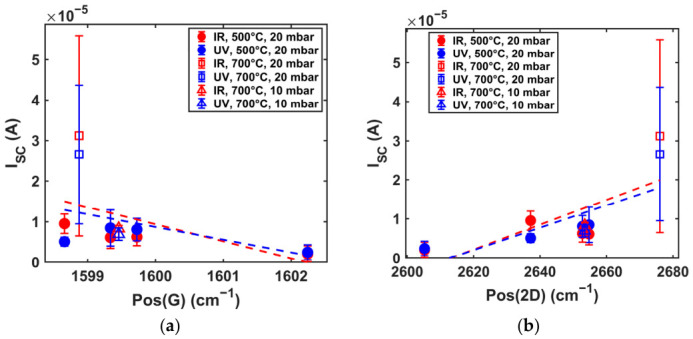
Short-circuit current vs. Pos(G) (**a**) and Pos(2D) (**b**).

The photocurrent increased with Pos(2D) upshift and decreased with Pos(G) upshift (Figure 6). Similarly to the photocurrent, the short-circuit current (I_SC_) increased with Pos(2D) upshift and decreased with Pos(G) upshift (Figure 7). The observed relation between Raman peak positions and photoelectric parameters suggests that graphene self-doping may be one of the factors affecting the photocurrent and short-circuit current. No clear dependence of the photocurrent and short-circuit current on the graphene layer number, defect density, or defect type was observed (Figures S4 and S5).

**Figure 8:**
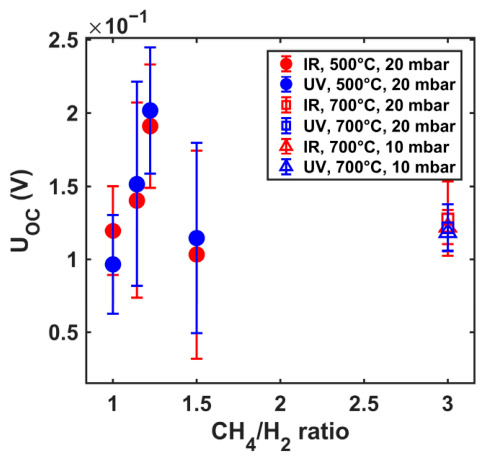
Open-circuit voltage vs. CH_4_/H_2_ gas flow ratio.

The open-circuit voltage was the highest for hydrogenated graphene synthesized at 500 °C using H_2_ and CH_4_ flow rates of 110 and 90 sccm, respectively (Figure 8). No clear dependence of the open-circuit voltage on Raman scattering spectra parameters was observed (Figure S6). Nevertheless, open-circuit voltage varied non-monotonically with the CH_4_/H_2_ gas flow ratio (Figure 8), with the highest value obtained at an intermediate gas flow ratio. In contrast, no clear dependence of the dark current, photocurrent or short-circuit current on the methane and hydrogen gas flow ratio was observed (Figure S7). Therefore, among the analyzed photovoltaic parameters, U_OC_ appears to be the most sensitive to the gas composition, although its variation cannot be assigned to a single Raman-derived structural parameter.

## 4. Discussion

In this research, consistent with our previous findings on low-temperature graphene synthesis on thermal SiO_2_ films [23], increasing the CH_4_/H_2_ flow ratio, along with a reduction in growth temperature from 700 °C to 500 °C, shifted the dominant defect types in the graphene. As discussed in the Raman analysis section, the low I(D)/I(D′) ratio, together with the suppressed 2D band and the enhanced D + D′ band, is consistent with the growth of hydrogenated graphene under the present low-temperature, high-methane-flow PECVD conditions. However, the type and density of graphene defects did not appear to influence graphene self-doping.

The decreased CH_4_/H_2_ flow ratio led to the growth of graphene with fewer layers. At the same time, enhanced self-doping was observed for samples grown at 500 °C using elevated CH_4_/H_2_ flow ratios. This may be related to the fact that the charge transfer from the substrate is strongest for layers closest to the interface and decreases with distance [46]. Notably, the self-doping in pristine graphene samples was lower than in most samples deposited at 500 °C, despite the fact that pristine graphene was synthesized using a significantly lower CH_4_/H_2_ ratio. This indicates that the observed self-doping cannot be attributed solely to the graphene layer number. Different hydrocarbon species can be adsorbed on the Si(100) surface [47], and some products of these reactions can remain stable at temperatures higher than those used in the present study for graphene growth [48]. In addition, graphene can be doped by placing various hydrocarbon molecules between the SiO_2_ substrate and graphene [49]. Thus, one may suppose that in the case of the hydrogenated graphene, the increased flux of incompletely dissociated hydrocarbon species may modify the silicon surface charge state, thereby enhancing charge transfer to graphene and increasing self-doping.

Regarding the growth mechanisms, an increased CH_4_/H_2_ ratio affected graphene synthesis conditions by increasing the flux of carbon-containing species required for graphene growth and reducing the relative influence of hydrogen-induced etching [43,44]. This adjustment enabled graphene growth at a lower temperature and increased the number of graphene layers. Additionally, the combination of increased methane flow and reduced process temperature made the dissociation of hydrocarbon molecules less efficient [50], thereby promoting the formation of hydrogenated graphene.

A comparative analysis of pristine graphene grown at 700 °C and samples grown at 500 °C indicates that a decrease in the synthesis temperature results in a rougher surface and, thereby, increased conductivity at the expense of morphological integrity. In this case, increasing the methane concentration acts as a stabilizing factor, restoring surface planarity and reducing maximum conductivity.

Furthermore, the dark reverse current in certain graphene/Si junctions grown at 500 °C was lower than that of pristine graphene/Si(100) junctions. Self-doping of graphene led to decreases in photocurrent and short-circuit current. Notably, it was shown by other authors that p-type doping of graphene increases the efficiency of graphene/n-Si solar cells by raising the barrier height at the graphene/Si junction [51]. Thus, in our case, the observed decrease in photocurrent and short-circuit current with graphene self-doping may be related to a reduction in the effective graphene/n-Si junction barrier. In this context, the highest photocurrent and I_SC_ were observed for the pristine graphene/Si junction grown at a working pressure of 20 mBar. However, these parameters for the pristine graphene/Si(100) junction grown at 10 mBar were lower than those of several samples synthesized at 500 °C.

On the other hand, the open-circuit voltage (U_OC_) reached its maximum for the hydrogenated graphene synthesized at 500 °C with flow rates of 110 sccm H_2_ and 90 sccm CH_4_ (Figure 8). Recombination at the graphene/Si interface is one of the main factors limiting the performance of graphene/Si solar cells [2]. Thus, a reduced surface recombination rate can result in an increased open-circuit voltage of the solar cell [51,52]. Therefore, proper passivation is crucial [2,53]. In particular, it should be noted that hydrogenated graphene has been employed as an interfacial passivating layer in the graphene/Si junction [54], and hydrogen passivation can stabilize graphene edges [55]. Thus, in our study, the observed weak increase in U_OC_ may be related to hydrogen-related passivation effects at the graphene/Si interface.

In such a way, one can conclude that the low-temperature PECVD growth conditions, especially the CH_4_/H_2_ ratio, strongly affect the graphene structure and contribute to some of the observed changes in the electrical and photoelectrical properties of the graphene/Si junctions.

## 5. Conclusions

In conclusion, direct PECVD growth of graphene on Si(100) at 500 °C was achieved by increasing the CH_4_/H_2_ gas flow ratio, compared with the conditions used for pristine graphene synthesis at 700 °C. Raman analysis showed that lowering the synthesis temperature and adjusting the gas flow ratio changed the dominant defect type from grain-boundary defects to hydrogen-related on-site defects, and the obtained Raman features were consistent with the growth of hydrogenated graphene under low-temperature, high-methane-flow PECVD conditions.

The structural properties of the graphene layers synthesized at 500 °C depended strongly on the CH_4_/H_2_ ratio. A lower methane-to-hydrogen gas flow ratio resulted in the growth of fewer graphene layers and stronger self-doping effects, while AFM/CAFM measurements showed that lowering the synthesis temperature led to rougher surfaces and increased local conductivity compared with pristine graphene grown at 700 °C. Increasing the methane concentration partially restored surface planarity and reduced the maximum local current. These effects of the CH_4_/H_2_ ratio on the growth process can be related to the interplay between the flux of carbon-containing species required for graphene growth and the influence of hydrogen-induced etching, while the combination of the increased methane flow and reduced process temperature makes the dissociation of hydrocarbon molecules less efficient and favors the formation of hydrogenated graphene.

The electrical and photoelectrical properties of the graphene/Si junctions were found to depend on the graphene structure. Graphene self-doping was associated with decreases in photocurrent and short-circuit current, which may be related to a reduction in the graphene/n-Si barrier height. In contrast, the open-circuit voltage reached its maximum for the hydrogenated graphene synthesized at 500 °C using 110 sccm H_2_ and 90 sccm CH_4_ flows, suggesting that hydrogen-related passivation effects may improve the graphene/Si interface. Thus, hydrogenated graphene synthesized directly on Si at 500 °C may be considered a viable low-temperature alternative to pristine graphene grown at 700 °C, especially for graphene/Si photodetectors and photovoltaic junctions.

Overall, the results show that low-temperature direct PECVD growth on Si allows tuning of the defect structure, layer number, self-doping, and interface-related properties of graphene and that these factors strongly influence the performance of graphene/Si photoactive junctions. At the same time, further optimization of the growth parameters remains necessary to maximize the photoelectric characteristics of the graphene/Si junctions.

## Figures and Tables

**Table 1:** The synthesis conditions used for sample growth.

Sample No.	Power, kW	CH_4_, sccm	H_2_, sccm	Pressure, mBar	Temperature, °C	Time, min
1	0.7	50	50	10	500	60
2	0.7	50	50	20	500	60
3	0.7	45	55	20	500	60
4	0.7	75	75	20	500	60
5	0.7	70	80	20	500	60
6	0.7	80	120	20	500	60
7	0.7	85	115	20	500	60
8	0.7	90	110	20	500	60
9	0.7	25	75	10	700	60
10	0.7	25	75	20	700	60

**Table 2:** Roughness, height, and conduction current parameters of the graphene layers.

CH_4_:H_2_	R_q_ (nm)	R_sk_	Current (pA)
25:75	0.17 ± 0.02	0.08 ± 0.01	26.7 ± 1.5
80:120	1.59 ± 0.15	0.75 ± 0.08	45.7 ± 3.2
90:110	0.33 ± 0.04	−0.09 ± 0.02	20.6 ± 1.1

## Data Availability

Data are contained within the article.

## Supplementary Materials

The following supporting information can be downloaded at: https://www.mdpi.com/article/10.3390/mi17070801/s1, Figure S1: Plots of the Raman scattering spectra parameters: I(D)/I(D’) vs. I(D)/I(G); (a); Pos(2D) vs. I(D)/I(D’) (b); Figure S2: The dependence of the I(D)/I(G) ratio (a) and I(D)/I(D’) ratio (b) on the methane and hydrogen gas flow ratio; Figure S3: Dark reverse current measured at −0.3 V vs. Raman scattering spectra parameters; Figure S4: Photocurrent vs. Raman scattering spectra parameters: I(D)/I(G) (a), I(D)/I(D’) (b) and I(2D)/I(G) (c) ratios; Figure S5: Short-circuit current vs. Raman scattering spectra parameters: I(D)/I(G) (a), I(D)/I(D’) (b) and I(2D)/I(G) (c) ratios; Figure S6: Open-circuit voltage vs. Raman scattering spectra parameters: Pos(G) (a), Pos(2D) (b) I(2D)/I(G) ratio (c), I(D)/I(G) ratio (d) and I(D)/I(D’) ratio (e); Figure S7: Dark reverse current (a), photocurrent (b) and short-circuit current (c) vs. CH4/H2 gas flow ratio.

## Abbreviations

The following abbreviations are used in this manuscript:

CVD	Chemical vapor deposition
PECVD	Plasma-enhanced chemical vapor deposition
FWHM	Full-width at half-maximum (FWHM)
Pos(G)	Position of G peak
Pos(2D)	Position of 2D peak
AFM	Atomic force microscopy
CAFM	Conductive atomic force microscopy
R_q_	Root mean square roughness
R_sk_	Skewness
I–V	Current–voltage
I_rd_	Dark reverse current
I_ph_	Photocurrent
I_SC_	Short-circuit current
U_OC_	Open-circuit voltage
