# Supplementary material for: Low-Temperature Direct PECVD Synthesis of Graphene on Si(100) with Increased Methane Flow: Structure and Photoelectric Properties
Source: Micromachines (Basel). 2026 Jun 30;17(7):801. doi: 10.3390/mi17070801 (PMC13413903; doi:10.3390/mi17070801)
Supplement: Supplementary file 1 [file micromachines-17-00801-s001.zip › micromachines-4355087-supplementary.pdf]

micromachines

MDPI

Article

Low-Temperature Direct PECVD Synthesis of Graphene on Si(100) with Increased Methane Flow: Structure and Photoelectric Properties

Vidmantas Kumža [1,*], Rimantas Gudaitis [1], Asta Guobienė [1], Andrius Vasiliauskas [1], and Šarūnas Meškinis [1,*]

[1] Institute of Materials Science, Kaunas University of Technology, K. Baršausko 59, LT-51423 Kaunas, Lithuania;

* Correspondence: vidmantas.kumza@ktu.lt (V.K.); sarunas.meskinis@ktu.lt (Š.M.)

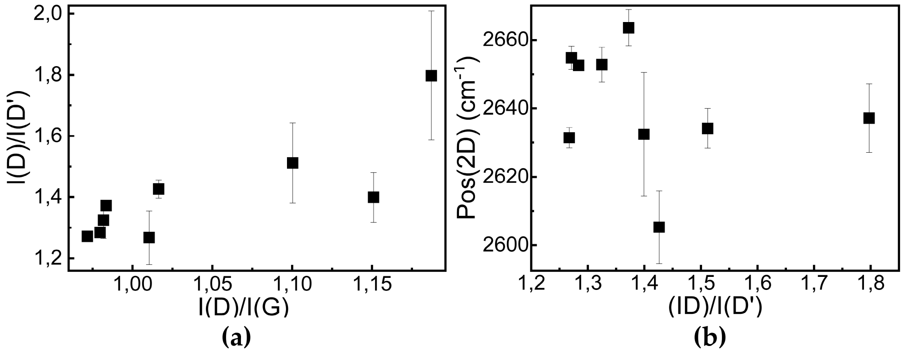

Figure S1. Plots of the Raman scattering spectra parameters: I(D)/I(D′) vs. I(D)/I(G); (a); Pos(2D) vs. I(D)/I(D′) (b).

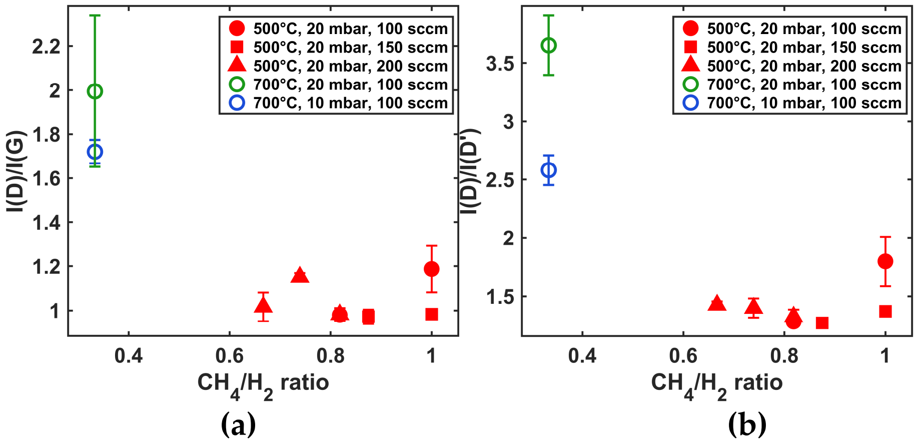

Figure S2. The dependence of the I(D)/I(G) ratio (a) and I(D)/I(D′) ratios (b) on methane and hydrogen gas flows ratio.

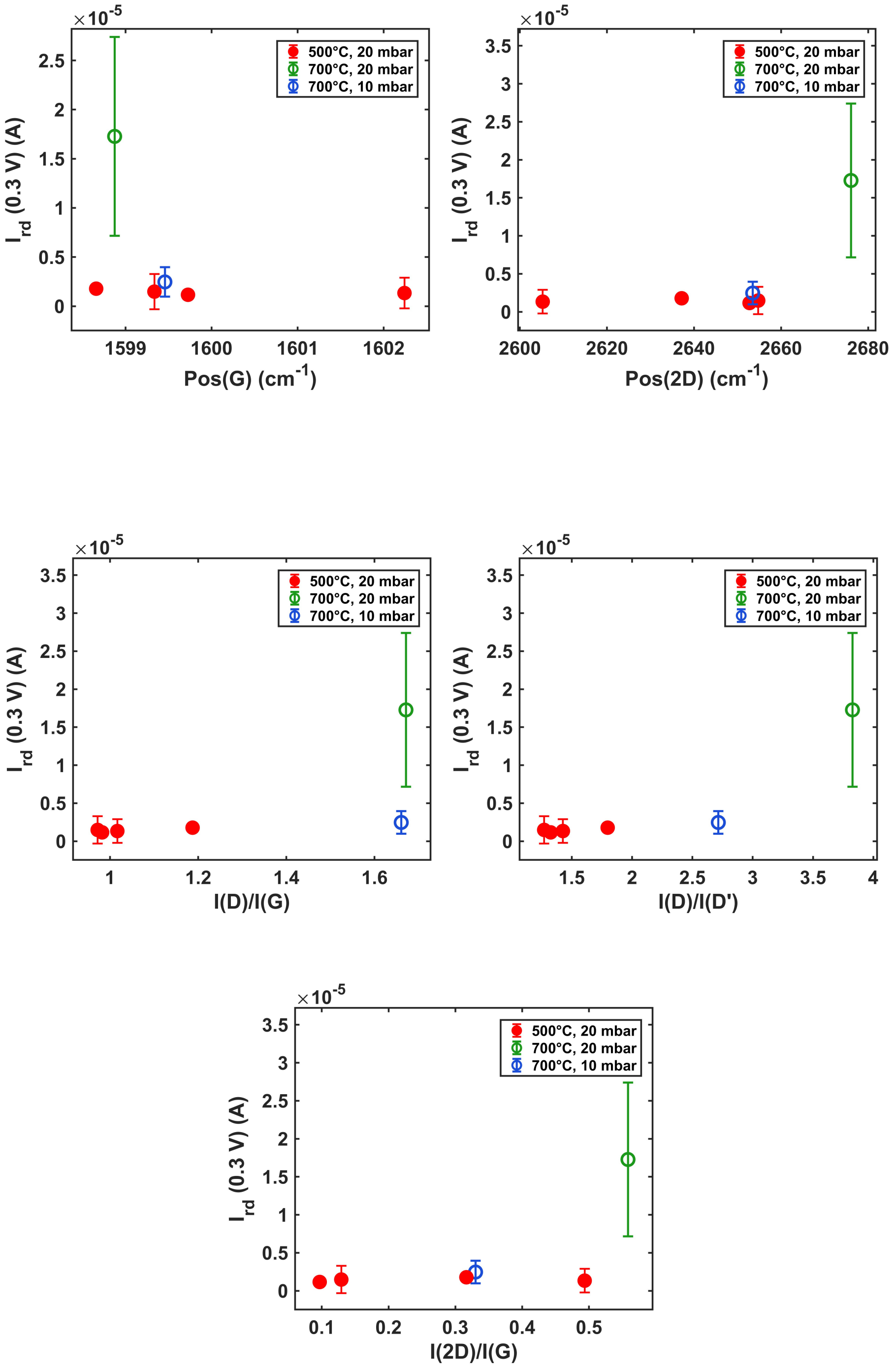

Figure S3. Dark reverse current measured at -0,3 V vs. Raman scattering spectra parameters.

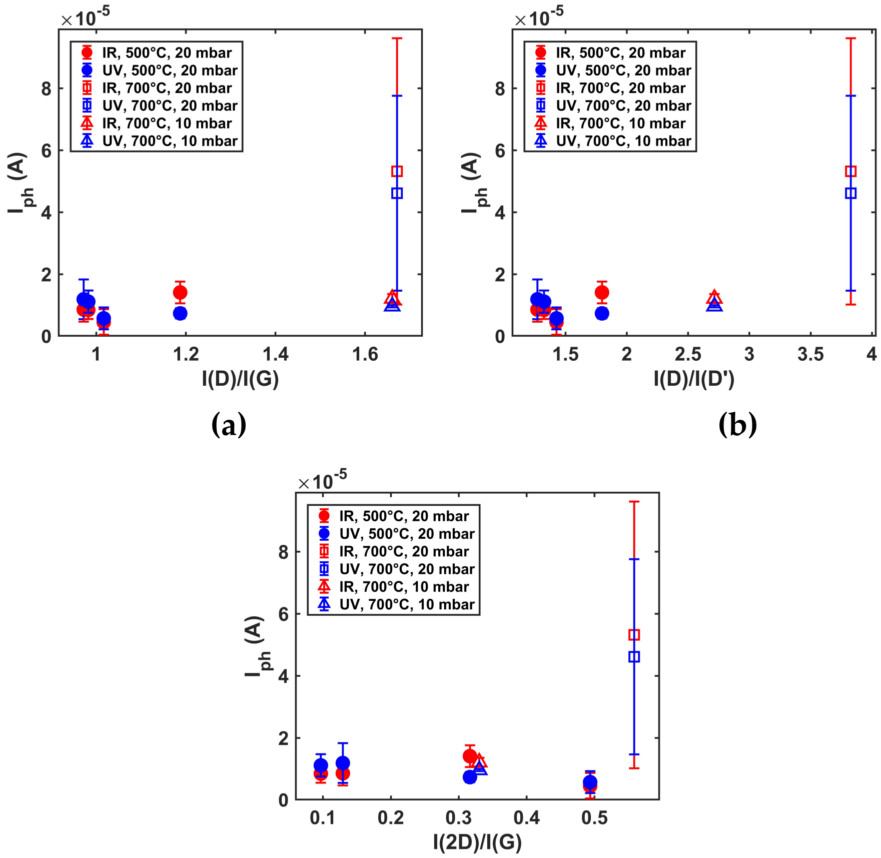

(a) **(b)**

(c)

Figure S4. Photocurrent vs. Raman scattering spectra parameters: I(D)/I(G) (a), I(D)/I(D′) (b) and I(2D)/I(G) (c) ratios.

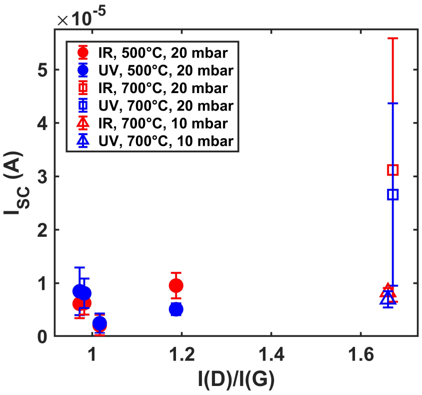

(a)

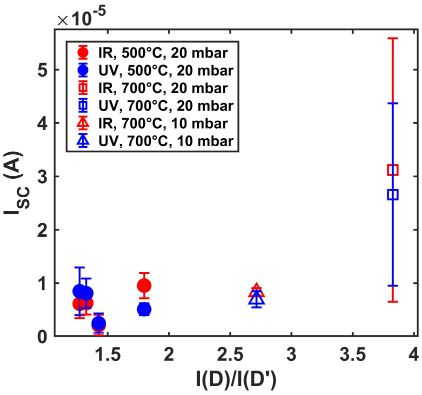

(b)

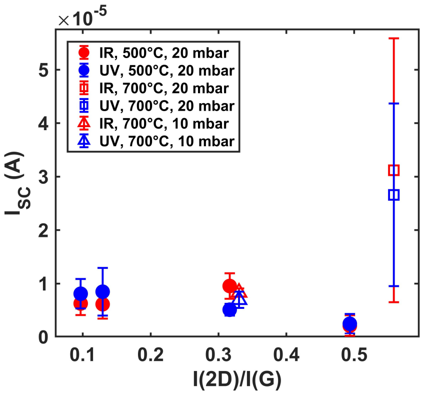

(c)

Figure S5. Short circuit current vs. Raman scattering spectra parameters: I(D)/I(G) (a), I(D)/I(D′) (b) and I(2D)/I(G) (c) ratios.

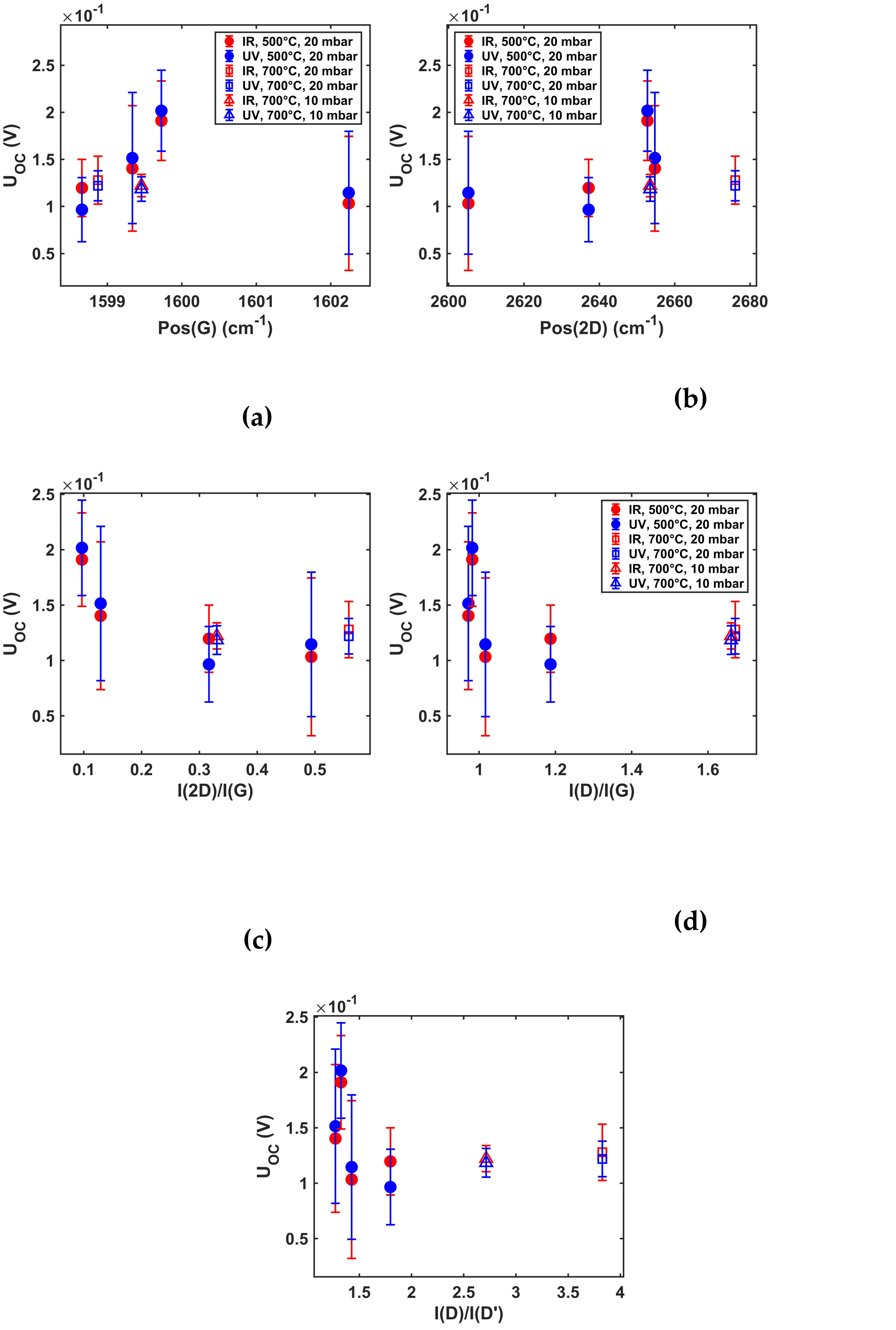

(e)

Figure S6. Open circuit voltage vs. Raman scattering spectra parameters: Pos(G) (a), Pos(2D) (b) I(2D)/I(G) ratio (c), I(D)/I(G) ratio (d) and I(D)/I(D′) ratio (e).

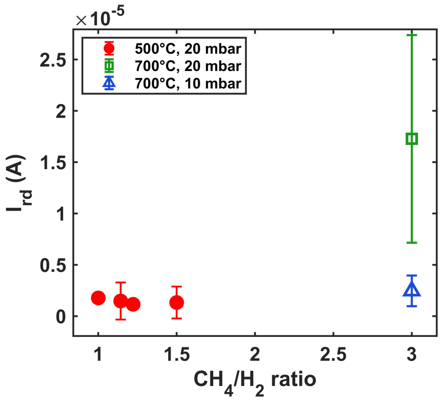

(a)

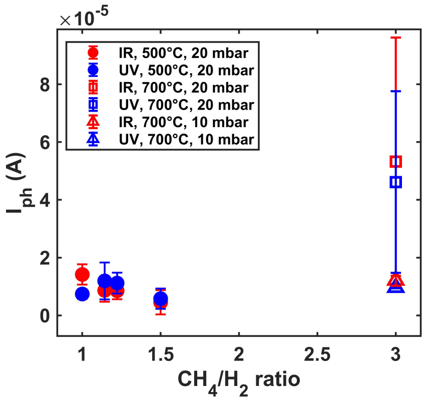

(b)

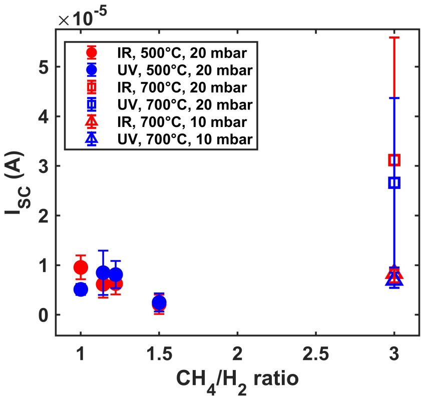

(c)

Figure S7. Dark reverse current (a), photocurrent (b) and short-circuit current (c) vs. CH_4/H_2 gas flow ratio.
